# Anti-Inflammatory Effects of Ginsenoside Rg1 and Low-Dose Ginseng Extract in an Astrocyte–Microglia Co-Culture Model of Inflammation

**DOI:** 10.3390/pharmaceutics18070806

**Published:** 2026-06-29

**Authors:** Shaoning An, Laura Schönfelder, Peter Reusch, Pedro M. Faustmann, Fatme S. Ismail, Timo Jendrik Faustmann

**Affiliations:** 1 Department of Neuroanatomy and Molecular Brain Research, Medical Faculty, Ruhr University Bochum, 44801 Bochum, Germany; shaoning.an@ruhr-uni-bochum.de (S.A.); pedro.faustmann@ruhr-uni-bochum.de (P.M.F.); 2 Department of Clinical Pharmacology, Medical Faculty, Ruhr University Bochum, 44801 Bochum, Germany; peter.reusch@ruhr-uni-bochum.de (P.R.); fatme.ismail@ruhr-uni-bochum.de (F.S.I.); 3 Department of Psychiatry and Psychotherapy, Medical Faculty, Heinrich Heine University Düsseldorf, 40225 Düsseldorf, Germany

**Keywords:** ginseng extract, ginsenoside Rg1, astrocyte, microglia, neuroinflammation

## Abstract

**Background**: Neuroinflammation contributes to the etiopathology and symptom severity of neurodegenerative and neuropsychiatric disorders. Glial cells, especially microglia and astrocytes, play a crucial role in neuroinflammation. It has been reported that ginseng (*Panax ginseng*) and its bioactive component ginsenoside Rg1 exhibit anti-inflammatory effects and can improve cognitive performance in various models. However, the exact underlying mechanisms remain unclear. **Methods**: Astrocyte–microglia co-culture models simulating physiological (M5, 5–10% microglia) and pathological/inflammatory (M30, 30–40% microglia) conditions were treated with different concentrations of ginsenoside Rg1 (15, 30, 45 µM) or ginseng extract (derived from Korean red ginseng) at low (12.5, 25, 37.5 µg/mL) or high doses (125, 250, 375 µg/mL) for 24 h. Cell viability was assessed using the MTT assay while microglial reactivity was examined using immunocytochemistry. Astrocytic gap-junctional coupling was investigated using the scrape-loading method, and connexin 43 (Cx43) expression was analyzed using immunocytochemistry and Western blot. **Results**: Both Rg1 and low-dose ginseng extract reduced microglial activation under inflammatory conditions by promoting a shift in microglia from an activated to homeostatic (resting) phenotype. Rg1 preserved astrocytic gap-junctional function by preventing the inflammation-induced downregulation of Cx43 expression and enhancing Cx43-mediated gap-junctional intercellular communication. Rg1 caused a significant reduction in glial cell viability, but only at high concentrations (30 and 45 µM), under inflammatory conditions. High-dose ginseng extract showed a significant concentration-dependent reduction in glial cell viability under physiological and pathological conditions, without comparable anti-inflammatory benefits. **Conclusions**: This study demonstrates that low-dose ginseng and its active compound Rg1 exert anti-inflammatory effects by modulating astrocytic coupling and microglial reactivity. These results provide a novel therapeutic perspective for the use of ginseng in the treatment of neurodegenerative and neuropsychiatric diseases related to neuroinflammation.

## 1. Introduction

Inflammation of the central nervous system (CNS) is a challenging condition that is implicated in the pathogenesis of various neurodegenerative and neuropsychiatric disorders, such as multiple sclerosis, stroke, epilepsy, meningoencephalitis, Alzheimer’s disease, depression and schizophrenia [1]. The condition of neuroinflammation is not clearly defined, but it does involve non-neuronal glial cells, e.g., astrocytes and microglia, the latter being the main resident immune cells of the brain [2,3,4,5]. Microglia are highly adaptive cells that can change their morphology in response to stimuli resembling inflammatory conditions—ranging from a homeostatic (previously referred to as resting) ramified type, through an intermediate type, to a rounded phagocytic (reactive) type [6]. Microglia can react to cytokines and promote inflammatory reactions, are involved in phagocytosis and synaptic pruning, and can undergo priming after exposure to early life stressors [7,8,9,10]. In addition, interactions between astrocytes and microglia regulate CNS processes in health and disease. Study findings suggest that bidirectional astrocyte–microglia communication modulates neuroinflammation through various pathways including the secretion of multiple cytokines and inflammatory mediators. Reactive microglia produce cytokines such as Interleukin-1 alpha/beta (IL-1 α/β) and tumor necrosis factor alpha (TNF-α), which lead to astrocytic reactivity. In response to this, reactive astrocytes release further pro-inflammatory mediators such as complement proteins and chemokines, perpetuating CNS inflammation. This microglia–astrocyte crosstalk has also been demonstrated in neurodegenerative diseases, where microglia-induced astrocytic reactivity drives the spread of neuroinflammation and synaptic dysfunction [11,12]. Taken together, microglia are an interesting target for pharmacological anti-inflammatory approaches to treat CNS disorders. We previously performed various pharmacological studies (using anticonvulsive drugs, mood-stabilizing drugs, glucocorticoids and immunomodulatory drugs) in an established astrocyte–microglia co-culture model, aiming to investigate the drugs’ effects on microglia-mediated inflammation [13,14,15,16,17]. Physiological and pathological conditions were modeled using microglia–astrocyte co-cultures: so-called “physiological M5 co-cultures” containing 5–10% microglial cells and “pathological, inflammatory M30 co-cultures” with 30–40% microglial cells [6]. There is a demand for new pharmacological approaches, with a popular substance in this context being ginseng extract, as well as its active component ginsenoside Rg1, derived from the medicinal herb *Panax ginseng*. Ginseng has been used in China for thousands of years to treat various medical conditions due to its beneficial and healing effects, e.g., boosting immune function, antifatigue effects and improving cognitive functions [18,19]. Air-drying, steaming and fermentation are typically performed to preserve and enhance the effectiveness of ginseng roots [20]. Ginsenoside Rg1 has been demonstrated to reduce depressive-like behavior and to regulate the hypothalamic–pituitary–adrenal axis and synaptic plasticity [21]. Moreover, neuroprotective effects in cerebral ischemia–reperfusion models [22,23], Alzheimer’s disease models [24,25] and spinal cord injury models [26], as well as the ability to promote neurite growth in retinal ganglion cells, have been reported for Rg1 [27], in addition to regulatory effects on astrocytes and microglia [28,29]. In contrast to ginsenoside Rg1, which is a single constituent with specific pharmacological targets, ginseng extract contains a complex mixture of multiple ginsenosides and other constituents [30]. Differences in Panax species, extraction procedures, manufacturing processes and storage conditions can lead to variations in the composition and distribution of ginsenosides in ginseng extract products [31,32]. Thus, we aimed to study the effects of ginsenoside Rg1 and low/high doses of ginseng extract in established M5 (physiological) and M30 (pathological, inflammatory) astrocyte–microglia co-culture models with regard to cell viability, microglial morphology, connexin 43 (Cx43) expression and gap-junctional intercellular communication.

## 2. Materials and Methods

### 2.1. Cell Culture

The astrocyte–microglia co-culture was prepared from brain hemispheres of postnatal 0- to 2-day-old (P0–P2) Wistar rats according to the protocol established by Faustmann et al. [6]. All experiments were approved by the local authorities in Bochum, Germany, and performed according to the ethical standards of the Ruhr University Bochum and the German Animal Welfare Act. Following decapitation of the rats, the meninges and choroid plexus were removed and the brain tissue was collected in ice-cold phosphate-buffered saline (PBS) (containing 1.38 M NaCl, 27 mM KCl, 81 mM NaH_2_PO_4_, and 14.7 mM K_2_H_2_PO_4_, with pH adjusted to 6.7 using 10 N NaOH (J.T. Baker, Deventer, The Netherlands)). The collected brain tissue was transferred into a 0.1% trypsin solution (PAA laboratories, Pasching, Austria) for 30 min at 37 °C for homogenization. The homogenate was then centrifuged at 500 g for 12 min at room temperature. The trypsin–PBS supernatant was discarded. Next, 4 mL of sterile astrocyte medium and 1 mL of DNase I (Serva Electrophoresis, Heidelberg, Germany) (100 µL/mL in Dulbecco’s minimal essential medium (DMEM; Invitrogen, Karlsruhe, Germany)) were added to initiate tissue digestion, and the mixture was incubated for 5 min at room temperature. The cell suspension was diluted to a total volume of 20 mL with washing medium (containing 10% fetal calf serum (FCS) and 1% penicillin/streptomycin solution (Biochrom AG, Berlin, Germany)) and centrifuged at 200 g for 5 min. A final centrifugation at 200 g for 5 min was performed after decantation of the washing medium and replacing it with 20 mL of fresh medium. The pellet was resuspended with 2 mL DMEM per brain and filtered through a nylon mesh (60 µm). The filtered cells (2 mL of suspension) were seeded into a plastic cell culture flask with 4 mL of astrocyte culture medium (containing 10% fetal calf serum (FCS), 1% non-essential amino acids, 1% glutamine, and 1% penicillin/streptomycin solution (PAA laboratories, Linz, Austria)) and incubated at 37 °C and 5% CO_2_. The following day, the cells were washed again under microscopic observation and cultured in fresh astrocyte medium. After approximately 4 to 5 days, the confluent cells were ready to be passaged or used for further experiments. Depending on the extent of manual shaking, microglia and oligodendrocytes located on the surface of astrocytes could be separated so that two different proportions of microglia in the co-cultures were generated, which contained 5–10% (M5, simulating physiological conditions) or 30–40% (M30, simulating pathological inflammatory conditions) microglia.

### 2.2. Treatment of Cultures

Based on previous studies on Rg1’s effects on cell viability in astrocytes and microglia under in vitro conditions [33,34,35], the astrocyte–microglia co-cultures were incubated with Rg1 (European Pharmacopoeia Reference Standard, EDQM, Strasbourg, France) at concentrations of 15, 30 or 45 µM or a comparable concentration of ginseng extract (*Panax ginseng*) derived from Korean red ginseng capsules (Korea Ginseng Vertriebs GmbH, Lohmar, Germany) (12.5, 25 or 37.5 µg/mL) for 24 h. In addition, 10-fold higher concentrations of ginseng extract (125, 250 and 375 µg/mL) were tested to determine the potential harmful overactivity in astrocyte–microglia co-cultures. According to supplier information, after the harvest, the ginseng root is steam-treated and ground. The extraction solvent of the Korean red ginseng root is ethanol 60%. The drug native extract ratio is 3–3.8 = 1. Ethanol is removed via the following production step. The ginseng extract was produced from Korean red ginseng capsules consisting of 500 mg of extract, 10 mg of talc and 20 mg of magnesium stearate (total amount capsule contents = 530 mg). The total amount of the ginsenosides was 8.3% within the extract (Rb1 = 1.92%, Rb2 = 1.16%, Rc = 1.56%, Rd = 0.98%, Re = 0.84%, Rf = 0.38%, Rg1 = 1.03% and Rg2 = 0.35% (expressed as ginsenoside Rg1 as standardized parameter)) and was similar to amounts in other publications using ginseng extract [36,37]. For an overview and the structural formula, see Yang et al., 2023 [21]. The ginseng extract was analyzed using chromatography to identify ginsenosides (chromatography data of the batch (10000287/01) used is provided in the Supplementary Materials). A stock solution of Rg1 was prepared by dissolving 4 mg of Rg1 powder in 20 µL of DMSO (Sigma-Aldrich, Steinheim, Germany) and 480 µL of PBS. The 500 mg capsules of ginseng extract were dissolved in PBS at a concentration of 250 mg/mL and diluted with the culture medium. According to the Rg1 content of ginseng extract (1.03%), concentrations of 12.5, 25 and 37.5 µg/mL ginseng extract contain approximately 0.129, 0.258 and 0.386 µg/mL Rg1, corresponding to approximately 0.16, 0.32 and 0.48 µM Rg1, respectively. Untreated co-cultures served as control groups in each experimental series.

### 2.3. MTT Assay

The MTT assay (Roche Applied Sciences, Penzberg, Germany) was performed to analyze the cell viability, proliferation and cytotoxicity of astrocyte–microglia co-cultures after treatment. For this purpose, 20,000 cells per well were seeded into a poly-L-lysine (PLL)-coated 96-well plate (BD Biosciences, Heidelberg, Germany). Incubation was performed with the concentrations of Rg1 and ginseng extract described above for 24 h at 37 °C and 7% CO_2_ until the cultures reached 90–100% confluency. After incubation and replacement of the culture medium, 10 µL of the MTT agent was added to each well and the plate was incubated for 4 h at 37 °C and 7% CO_2_ to allow for the formation of crystals. Then, 100 µL of a solubilization solution was added to the co-cultures, which were then incubated overnight. The next day, the absorbance was measured photometrically at 550 nm using an Infinite 200 Pro microplate reader (Tecan Group Ltd., Männedorf, Switzerland).

### 2.4. Immunocytochemistry

**Figure 1:**
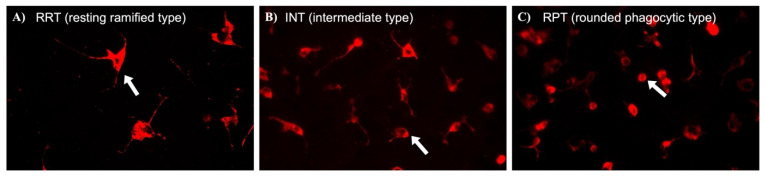
The immunocytochemistry of microglia morphology in astrocyte–microglia co-cultures at a magnification of 400×. Based on the morphology when stained with ED-1 antibody (red), three microglial phenotypic states were identified (white arrows): homeostatic (previously referred to as “resting”) ramified (**A**), intermediate (**B**) and rounded phagocytic (**C**).

Immunocytochemistry was used to count astrocytes and microglia, determine their phenotypes, and analyze the expression and localization of Cx43. Co-cultures were seeded (70,000 cells per well) onto coverslips (Assistant, Sondheim, Germany) in a 24-well plate. After incubation with the different concentrations of Rg1 and ginseng extract, the co-cultures were washed with PBS, fixed with ice-cold ethanol (Merck, Darmstadt, Germany) and washed again with PBS. To prevent non-specific binding between antigen epitopes and primary antibody, the co-cultures were incubated with a blocking reagent containing PBS, 1% bovine serum albumin (BSA) and 10% horse serum (HS) (PAA laboratories, Linz, Austria) for 1 h. After removing the blocking solution, the primary antibodies mouse anti-ED1 (Serotec, Düsseldorf, Germany) and rabbit anti-Cx43 (Invitrogen, Karlsruhe, Germany)), which were diluted in the blocking reagent (1:250 and 1:1000, respectively), were added (25 µL to each well). After a 24-h incubation period at 4 °C, the co-cultures were washed with PBS + 1% BSA and incubated with secondary antibodies (Alexa Red 568 anti-mouse and Alexa Green 488 anti-rabbit (Invitrogen, Karlsruhe, Germany)) diluted with blocking reagent (1:500) (25 µL per well) for 1 h. After 3 washes with PBS, the coverslips were mounted with ProLong Gold (Invitrogen, Karlsruhe, Germany) and 3 µL of DAPI (to label cell nuclei) onto slides. After overnight storage at 4 °C, the evaluation was conducted using a fluorescence microscope at 400X magnification. Three random visual fields per slide were evaluated for statistical analysis. DAPI staining was used to determine the total number of cells in the cultures, while ED1 staining was used to quantify the number of microglia. The microglial phenotypes were determined based on their distinct morphological characteristics with ED1 staining [6]. The homeostatic (“resting”) ramified type (RRT) of microglia exhibited small cell bodies with fine and ramified processes (Figure 1A); intermediate microglia (INT) showed shortened processes relative to their cell body (Figure 1B); and reactive (“activated”) microglia (RPT) displayed a round morphology without visible processes (Figure 1C). For quantitative analysis, microglia were categorized into two groups: RPT and microglia with processes including both RRT and INT phenotypes. This classification strategy was chosen to reduce potential errors associated with manual morphological classification and to improve the accuracy of phenotype assessment. Astrocytic Cx43 protein was immunologically visualized using anti-Cx43 antibody to determine its expression and localization. Cx43 expression was assessed by quantifying the percentage of Cx43-positive fluorescent area within each visual field.

### 2.5. Immunoblot (Western Blot) Analysis

To quantify astrocytic Cx43 expression, the co-cultures were seeded at 100,000 cells/well in a 6-well plate and incubated with the different concentrations of Rg1 and ginseng extract. After removing the medium, 200 µL of lysis buffer was added into each well to avoid protein degradation. Cell scrappers (Sarstedt, Nürnbrecht, Germany) were used to mechanically agitate the lysate and to detach the cells. The prepared samples were loaded into a 10% or 15% SDS–polyacrylamide gel (AppliChem, Darmstadt, Germany) and separated using electrophoresis at 100 volts for 20 min and then 150 volts for 1 h. After transferring the proteins to a nitrocellulose membrane (Amersham Pharmacia, Little Chalfont, England), the membrane was incubated in 2 mL of Odyssey blocking buffer (LI-COR Bioscience, Bad Homburg, Germany) for 1 h to prevent non-specific binding. After removal of the blocking buffer, the membrane was incubated with primary antibodies (rabbit anti-GAPDH (1:2000) (Sigma, St. Louis, MO, USA); rabbit anti-Cx43 (1:2000) (Sigma, St. Louis, MO, USA)) diluted in the blocking buffer. After incubation overnight at 4 °C and three washes with PBS–Tween (containing 0.05% (*v*/*v*) Tween 20 (AppliChem, Darmstadt, Germany)) for 20 min, the membrane was incubated with a secondary antibody (goat anti-rabbit IgG HRP (1:10,000) (Invitrogen, Karlsruhe, Germany)) for 1 h. The membrane was washed three times with PBS-Tween and once with PBS. After incubation with 2 mL of a reagent solution for 3 min, the membrane was scanned using a LI-COR Odyssey scanner and its associated software (LI-COR Bioscience, Bad Homburg, Germany). Cx43 expression was quantified using the ImageJ software (1.54 for Mac) by comparing it with the band of the housekeeping GAPDH.

### 2.6. Scrape-Loading–Dye Transfer Method

Scrape-loading was employed to investigate effects of different concentrations of Rg1 and ginseng extract on functional coupling and gap-junctional intercellular communication. The principle of scraping loading is based on the characteristics of Lucifer yellow dye transfer. Lucifer yellow cannot enter intact cell membranes, but it can diffuse through damaged membranes and be transported to neighboring cells through gap junctions, mainly Cx43-based gap junctions in astrocytes. Co-cultures were seeded (70,000 cells per well) onto coverslips in a 24-well plate. After 24 h of incubation with the different concentrations of Rg1 and ginseng extract, the co-cultures were washed with PBS and a modified insulin syringe (BD Bioscience, Heidelberg, Germany) with an injection needle (0.45 × 12 mm) (Braun, Melsungen, Germany) was employed to make a linear incision across the confluent cell layer on each coverslip. Next, 400 µL of a 0.03% (*w*/*v*) Lucifer yellow CH solution (in PBS) (Sigma-Aldrich, Steinheim, Germany) was added to each well and incubated for 3 min. Each well was then washed with PBS and immediately evaluated under a microscope. The functional coupling was evaluated using an Axiovert 100M fluorescence microscope (Carl Zeiss GmbH, Jena, Germany) at 100× magnification. Three random visual fields near the incision site per coverslip were evaluated. The fluorescent intensities of Lucifer yellow dye transfer were quantified as percentages using ImageJ software.

### 2.7. Data Analysis and Statistics

GraphPad Prism version 10 for Mac (GraphPad Software, San Diego, CA, USA) was employed for statistical analysis of the data. The normality of the data distribution was assessed using the Kolmogorov–Smirnov and D’Agostino–Pearson omnibus tests. One-way analysis of variance (one-way ANOVA) followed by Dunnett’s multiple comparisons test or Kruskal–Wallis test was used to compare two groups with normal distributions. Variance homogeneity was assessed using the Brown-Forsythe test. The statistical significance was set at *p* < 0.05, and the results are expressed as the mean ± standard error of the mean.

## 3. Results

### 3.1. Effects of Ginsenoside Rg1 and Ginseng Extract on Glial Cell Viability

**Figure 2:**
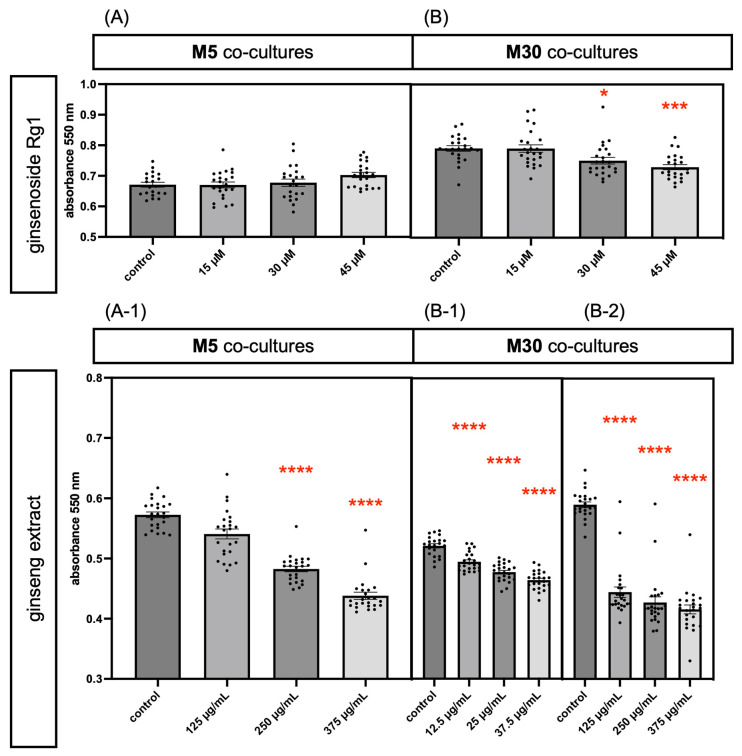
Glial cell viability assessed via the MTT assay in physiological (M5) (**A**,**A-1**) and pathological (M30) (**B**,**B-1**,**B-2**) co-cultures following 24 h of incubation with Rg1 (15–45 µM) or ginseng extract (12.5–375 µg/mL). No significant Rg1-dependent effects on cell viability were detected in M5 cultures (**A**) (n = 24). M30 co-cultures showed a significant reduction in glial cell viability following Rg1 treatment (**B**) (n = 24). Ginseng extract induced a significant reduction in glial cell viability in both M5 and M30 cultures (**A-1**,**B-1**,**B-2**) (n = 24). Untreated co-cultures served as controls. n refers to the number of analyzed wells for each treatment concentration. Statistical comparisons between individual groups with normality distribution test were performed using one-way analysis of variance (one-way ANOVA) followed by Dunnett’s multiple comparisons test or Kruskal–Wallis test: * *p* < 0.05, *** *p* < 0.001, and **** *p* ≤ 0.0001.

After a 24-h incubation period with Rg1, no significant changes in cell viability were observed in physiological M5 co-cultures (Figure 2A) (n = 24). In contrast, pathological M30 co-cultures exhibited a significant reduction in cell viability, from an absorbance rate of 0.79 in the control group (untreated cultures) to 0.75 at 30 µM Rg1 (*p* = 0.0202) and 0.73 at 45 µM Rg1 (*p* = 0.0002) (Figure 2B) (n = 24). In M30 co-cultures incubated with 12.5, 25 and 37.5 µg/mL ginseng extract, the glial cell viability exhibited a significant concentration-dependent reduction (Figure 2B-1) (n = 24; *p* ≤ 0.0001). In addition, incubation with 10-fold higher concentrations of ginseng extract (250 and 375 µg/mL) caused significant decreases in glial cell viability in both M5 and M30 cultures compared with the control group (Figure 2A-1,B-2) (n = 24; *p* ≤ 0.0001). Together, these results indicate that Rg1 and low-dose ginseng extract are associated with reduced cell viability under pathological conditions, whereas high-dose ginseng extract reduces cell viability under both physiological and pathological conditions.

### 3.2. Effects of Ginsenoside Rg1 and Ginseng Extract on Number of Cells Under Physiological and Pathological Conditions

**Figure 3:**
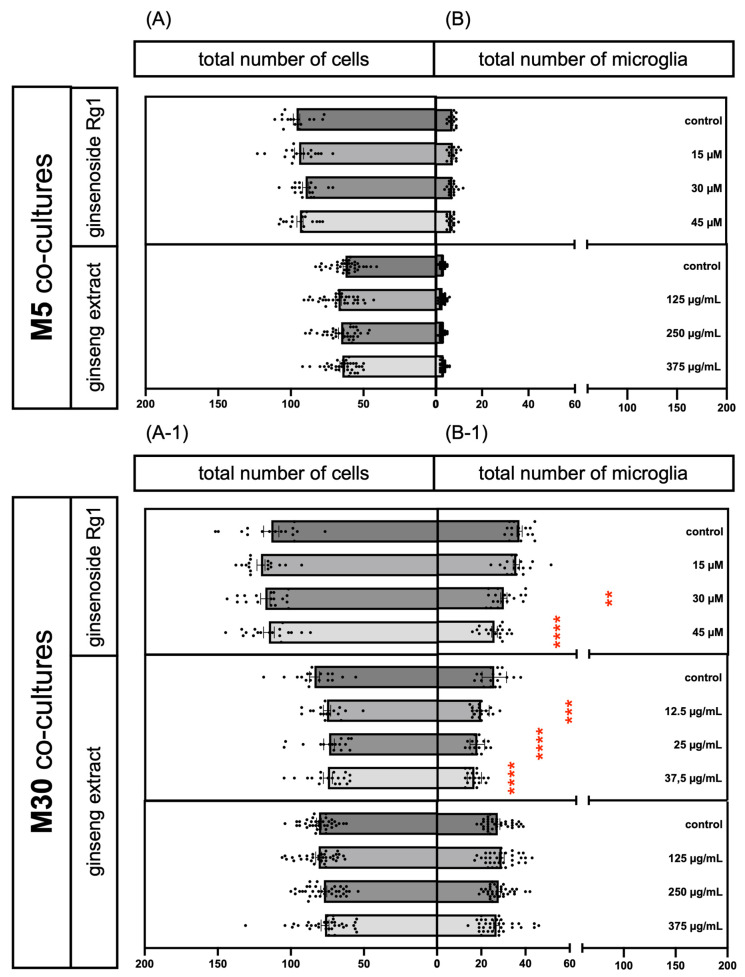
Total number of cells (**A**,**A-1**) and microglia (**B**,**B-1**), determined using immunocytochemistry, in physiological (M5) and pathological (M30) co-cultures following a 24-h incubation period with Rg1 or ginseng extract. Cell counts are presented as the number of cells per microscopic field (cells/field). Rg1 did not induce significant changes in total cell count in M5 cultures (**A**,**B**) (n = 18). In contrast, M30 co-cultures showed a significant concentration-dependent reduction in microglial cell numbers after treatment with Rg1 and low-dose ginseng extract (**B-1**) (n = 18). High-dose ginseng extract did not significantly alter cell counts in either M5 or M30 cultures (**A**,**A-1**,**B**,**B-1**) (n = 36). Untreated co-cultures served as controls. n refers to the number of microscopic fields analyzed from different wells per concentration. Statistical comparisons with normality distribution test were performed using one-way analysis of variance (one-way ANOVA) followed by Dunnett’s multiple comparisons test: ** *p* < 0.01, *** *p* < 0.001, and **** *p* ≤ 0.0001.

Following a 24-h incubation period with Rg1, no significant changes in total cell count or total number of microglia were observed in physiological M5 cultures (Figure 3A,B) (n = 18). In contrast, pathological M30 co-cultures showed a significant reduction in microglial cell numbers, from 35.375 in the control group to 28.611 at 30 µM Rg1 (*p* = 0.0013) and 24.611 at 45 µM Rg1 (Figure 3B-1) (n = 18). A significant concentration-dependent reduction in microglial cell counts was also observed in pathological M30 cultures treated with low-dose ginseng extract (Figure 3B-1) (n = 18; *p* < 0.001, *p* ≤ 0.0001). Incubation with high-dose ginseng extract did not induce significant changes in cell counts in either M5 or M30 cultures (Figure 3A,A-1,B,B-1) (n = 36). Taken together with the findings of Section 3.1, these results indicate that Rg1 and low-dose ginseng extract reduce pathological microglial numbers, whereas high-dose ginseng extract affects cell viability without changing total cell or microglial counts.

### 3.3. Effects of Ginsenoside Rg1 and Ginseng Extract on Microglial Activation Under Physiological and Pathological Conditions

**Figure 4:**
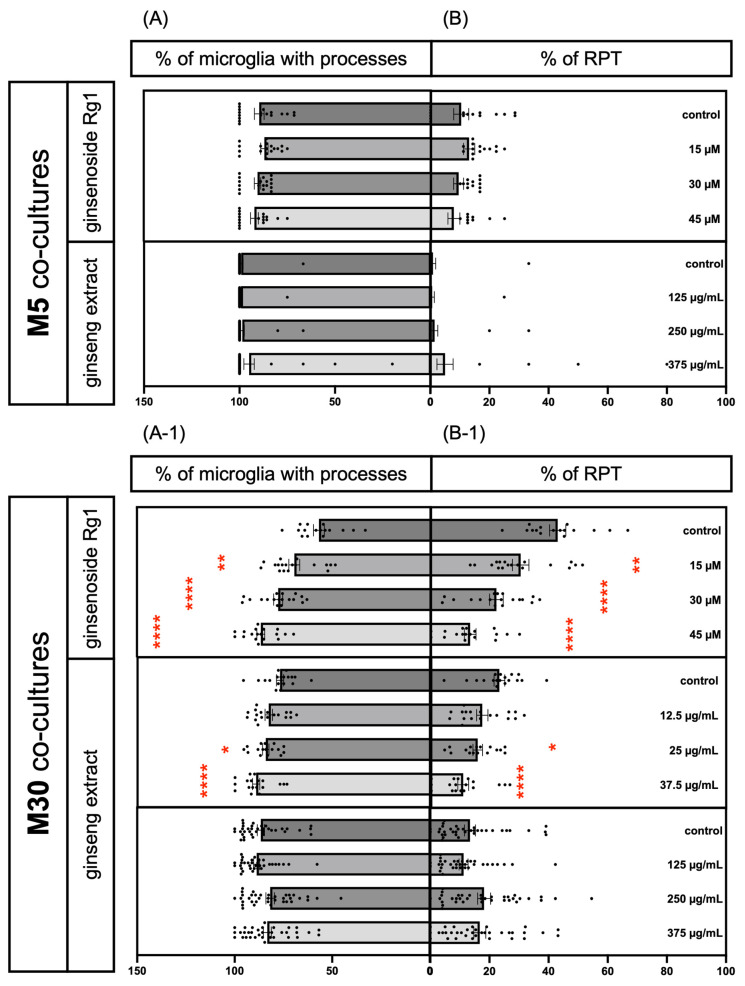
Microglial phenotypes, assessed via immunocytochemistry, in physiological (M5) and pathological (M30) co-cultures after a 24-h incubation period with Rg1 or ginseng extract. Rg1 did not alter microglial phenotypes in M5 cultures (**A**,**B**) (n = 18). In M30 co-cultures, both Rg1 and low-dose ginseng extract induced a significant concentration-dependent reduction in activated rounded phenotypes (RPT), which was accompanied by a significant increase in the percentage of microglia with processes (**A-1**,**B-1**) (n = 18). High-dose ginseng extract did not significantly impact microglial phenotypes in either M5 or M30 cultures (**A**,**A-1**,**B**,**B-1**) (n = 36). Untreated co-cultures served as control groups. n refers to the number of microscopic fields analyzed from different wells per concentration. Statistical comparisons with normality distribution test were performed using one-way analysis of variance (one-way ANOVA) followed by Dunnett’s multiple comparisons test or Kruskal–Wallis test: * *p* < 0.05, ** *p* < 0.01, and **** *p* ≤ 0.0001.

After a 24-h incubation period with Rg1, no significant changes in microglial phenotypes were observed in physiological M5 cultures (Figure 4A,B) (n = 18). In contrast, in pathological M30 cultures, Rg1 led to a significant concentration-dependent decrease in the proportion of rounded activated microglia (RPT), which was accompanied by a concentration-dependent increase in the percentage of microglia exhibiting processes (Figure 4A-1,B-1) (n = 18; *p* < 0.01, *p* ≤ 0.0001). Low-dose ginseng extract similarly showed a significant decrease in the proportion of activated microglia in pathological M30 cultures, from 23.11% in the control group to 15.78% at 25 µg/mL and 10.86% at 37.5 µg/mL (Figure 4B-1) (n = 18; *p* < 0.05, *p* ≤ 0.0001), which was accompanied by a significant increase in the percentage of microglia with processes, from 76.89% in the control group to 84.21% at 25 µg/mL and 89.13% at 37.5 µg/mL (Figure 4A-1) (n = 18; *p* < 0.05, *p* ≤ 0.0001). Incubation with high-dose ginseng extract did not result in significant effects on microglial phenotypes in either M5 or M30 cultures (Figure 4A,A-1,B,B-1) (n = 36). Together, these results demonstrate that Rg1 and low-dose ginseng extract suppress microglial activation and promote a shift from activated to the ramified phenotype, where high-dose ginseng extract does not exert comparable effects on microglial morphology.

### 3.4. Effects of Ginsenoside Rg1 and Ginseng Extract on Connexin 43 Expression and Localization Under Physiological and Pathological Conditions

**Figure 5:**
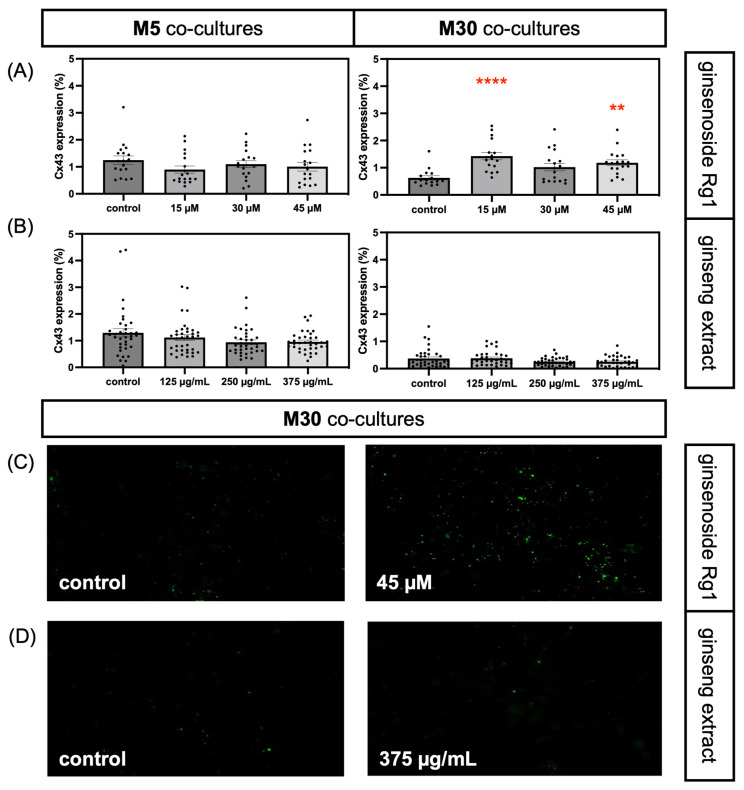
Astrocytic connexin 43 (Cx43) expression and localization, detected via immunocytochemistry, in physiological (M5) and pathological (M30) cultures after 24 h of incubation with Rg1 (**A**,**C**) or ginseng extract (**B**,**D**). Rg1 treatment did not significantly alter Cx43 expression in M5 cultures. In contrast, M30 co-cultures showed a significant increase in Cx43 expression following Rg1 treatment (**A**) (n = 18). High-dose ginseng extract did not change Cx43 expression in either M5 or M30 cultures (**B**) (n = 36). The effect of Rg1 is shown in the representative images, where treatment with 45 µM Rg1 increased Cx43 expression and its localization compared to the control in M30 cultures (**C**). Treatment with ginseng extract did not result in visible differences in Cx43 expression (light green) between control and treated group at 375 µg/mL in M30 cultures (**D**). Untreated co-cultures served as control groups. n refers to the number of microscopic fields analyzed from different wells per concentration. Statistical comparisons with normality distribution test were performed using one-way analysis of variance (one-way ANOVA) and Kruskal–Wallis test: ** *p* < 0.01, and **** *p* ≤ 0.0001.

Treatment with Rg1 did not result in significant alterations in Cx43 expression in physiological M5 cultures, as detected via immunocytochemistry (Figure 5A) (n = 18). In contrast, M30 co-cultures displayed a significant enhancement in Cx43 expression, quantified as the percentage of Cx43-positive fluorescence area per visual field, from 0.62% in the control group to 1.42% at 15 µM and 1.18% at 45 µM (*p* = 0.0015) (Figure 5A) (n = 18). Although the peak at 15 µM followed by a dip at 30 µM (*p* = 0.0576) created a U-shaped curve, the overall response was still increased expression of Cx43 compared with the control group. Incubation with high-dose ginseng extract did not lead to significant changes in Cx43 expression in either M5 or M30 cultures (Figure 5B) (n = 36). The representative immunocytochemical images in Figure 5C,D show the Cx43 expression and localization in the control and treated M30 cultures.

**Figure 6:**
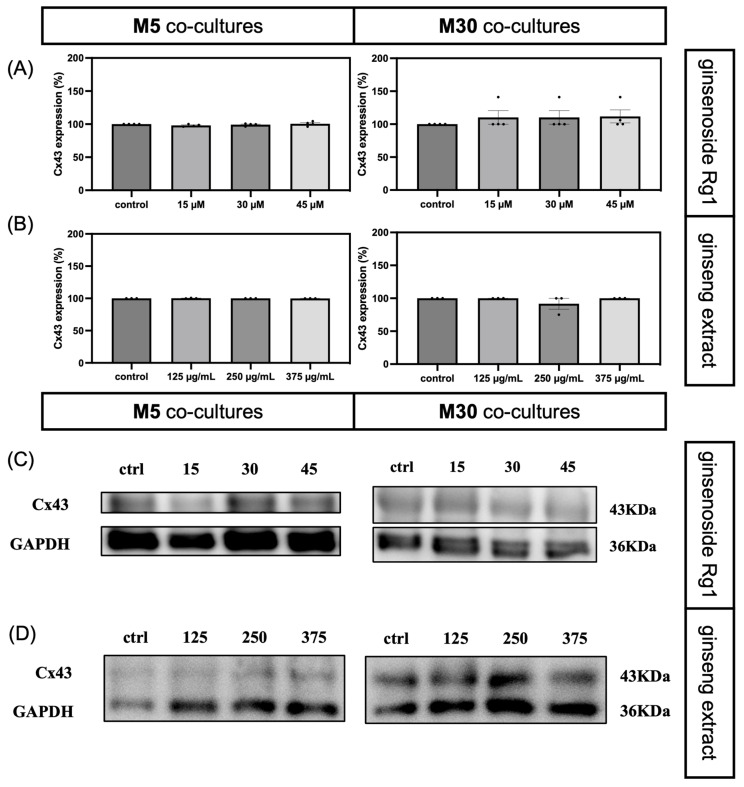
The expression of astrocytic connexin 43 (Cx43), assessed via Western blot analysis, in physiological (M5) and pathological (M30) cultures after a 24-h incubation period with Rg1 (**A**,**C**) or ginseng extract (**B**,**D**). Rg1 treatment did not significantly impact Cx43 expression in physiological M5 co-cultures. In M30 co-cultures, Rg1 showed a trend toward increased Cx43 expression but it did not reach statistical significance (**A**) (n = 4). High-dose ginseng extract did not alter Cx43 expression in either M5 or M30 cultures (**B**) (n = 3). The densities of immunoreactive bands also show effects of Rg1 and ginseng extract on Cx43 expression in both M5 and M30 cultures (**C**,**D**). Untreated co-cultures served as control groups. n refers to the number of independent experiments per concentration. Statistical comparisons with normality distribution test were performed using one-way analysis of variance (one-way ANOVA) followed by Dunnett’s multiple comparisons test or Kruskal–Wallis test.

In the Western blot analysis, no significant changes in Cx43 expression, quantified as the densities of immunoreactive bands, were observed following Rg1 treatment in physiological M5 cultures (Figure 6A,C) (n = 4). In M30 cultures, Rg1 treatment showed a trend toward increased Cx43 expression, although this did not reach statistical significance (Figure 6A,C) (n = 4). In contrast to the effects of Rg1, ginseng extract did not alter Cx43 expression in either M5 or M30 co-cultures (Figure 6B,D) (n = 3).

Together, these results indicate that Rg1 enhances Cx43 expression under pathological conditions via immunocytochemistry, despite displaying a U-shaped dose–response pattern with a reduction at 30 µM. In contrast, high-dose ginseng extract did not alter Cx43 expression via immunocytochemistry and immunoblotting.

### 3.5. Influence of Ginsenoside Rg1 and Ginseng Extract on Astrocytic Gap-Junctional Intercellular Communication Under Physiological and Pathological Conditions

**Figure 7:**
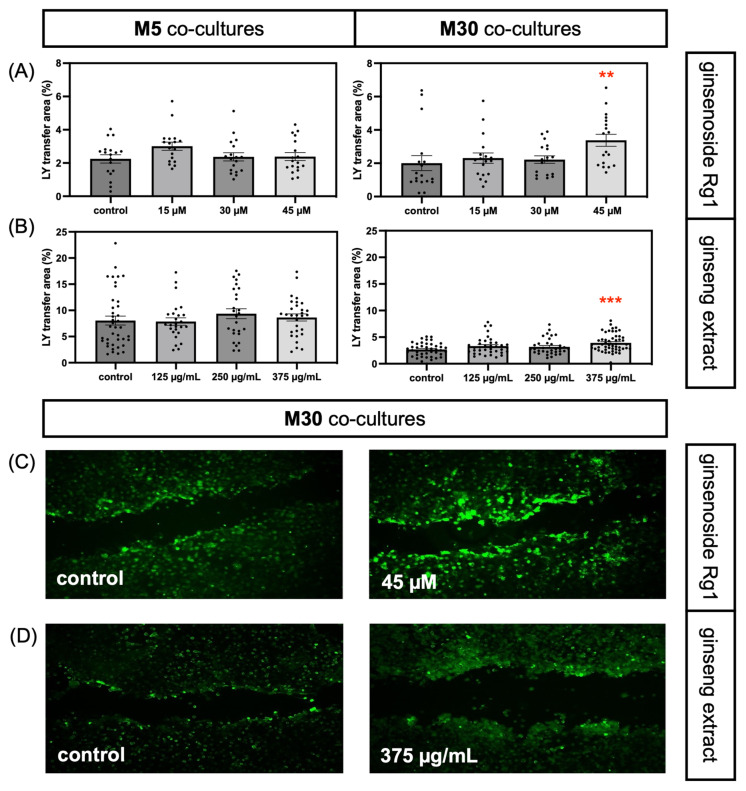
Astrocytic gap-junctional intercellular communication, detected using the scrape-loading–Lucifer Yellow (LY) dye transfer method, in physiological (M5) and pathological (M30) co-cultures after a 24-h incubation period with Rg1 (**A**,**C**) or ginseng extract (**B**,**D**). In physiological M5 co-cultures, Rg1 treatment did not significantly change the LY dye transfer area (**A**). In contrast, in pathological M30 cultures, a significant increase in LY dye transfer area was observed with Rg1 at 45 µM (**A**) (n = 18). High-dose ginseng extract did not significantly affect the gap-junctional intercellular communication in M5 cultures (**B**). However, in M30 co-cultures, treatment with 375 µg/mL ginseng extract significantly increased the LY dye transfer area (**B**) (n = 26). These effects are also shown in the representative images of M30 cultures, where both Rg1 and high-dose ginseng extract treatments visibly enhanced the LY dye transfer area compared to the control group (**C**,**D**). Untreated co-cultures served as control groups. n refers to the number of microscopic fields analyzed from different wells per concentration. Statistical comparisons with normality distribution test were performed using one-way analysis of variance (one-way ANOVA) and Kruskal–Wallis test: ** *p* < 0.01, *** *p* < 0.001.

After incubation with Rg1 for 24 h, no significant changes in gap-junctional intercellular communication were observed in physiological M5 cultures (Figure 7A) (n = 18). In contrast, M30 cultures showed a significant increase in the Lucifer Yellow dye transfer area at 45 µM Rg1, from 2.00% in the control group to 3.37% (*p* = 0.0018) (Figure 7A) (n = 18). High-dose ginseng extract did not significantly affect the gap-junctional intercellular communication in M5 cultures (Figure 7B) (n = 26). However, in M30 cultures, ginseng extract induced a significant increase in the Lucifer Yellow dye transfer area, from 2.68% in the control group to 3.92% at 375 µg/mL (*p* = 0.001) (Figure 7B) (n = 26). The representative images in Figure 7C,D show the effects of Rg1 and ginseng extract treatments compared to the control group in M30 cultures. Taken together, Rg1 and high-dose ginseng extract enhance astrocytic gap-junctional intercellular communication under pathological conditions.

## 4. Discussion

**Figure 8:**
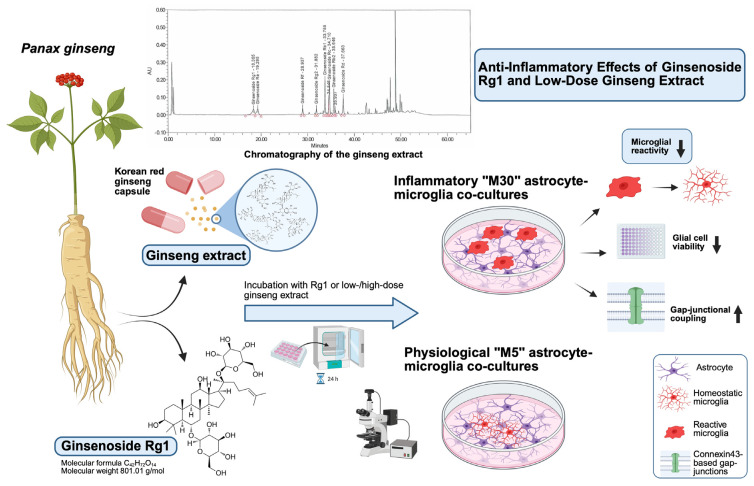
Graphical abstract summarizing main results (for details see Section 3).

In this study, we investigated the anti-inflammatory potential of ginseng and its bioactive component ginsenoside Rg1 in an astrocyte–microglia co-culture model. Our results demonstrate that, under pathological inflammatory conditions, both Rg1 and low/moderate concentrations of ginseng extract inhibited microglial proliferation and activation, which were accompanied by an increase in microglia with ramified phenotypes. Moreover, Rg1 enhanced astrocytic gap-junctional intercellular communication and connexin 43 expression. In contrast, high-dose ginseng extract significantly reduced cell viability but did not impact microglial activation or astrocyte Cx43 expression under pathological conditions (Figure 8).

In our initial experiments, Rg1 did not impact cell viability under physiological conditions, and the total number of cells remained unchanged. This is consistent with previous studies [33] showing that Rg1 does not exert astrocytic death, even at concentrations up to 100 µM. These findings correspond with our conclusion that Rg1 does not impair astrocyte-dominated cultures under physiological conditions. Similarly, another study reported that microglial viability remains unaffected by Rg1 at concentration of 0.5, 1 and 10 µM [34], which corresponds with our observation of stable microglial numbers in M5 cultures, even with higher concentrations of 15, 30 and 45 µM. In contrast to the results obtained under physiological conditions, pathological M30 cultures exhibited a significant Rg1 concentration-dependent reduction in both cell viability and microglial numbers following Rg1 treatment. These results suggest that Rg1 reduces pathological microglial proliferation and may restore normal levels of microglial viability. In other experiments using, e.g., lipopolysaccharide-induced microglia activation in BV2 microglia it was discussed that this effect may be reduced by Rg1-mediated activation of the microglial surface G-protein-coupled estrogen receptor [38,39,40].

Low-dose ginseng extract showed comparable effects to Rg1 under pathological conditions, significantly reducing cell viability and microglial numbers. In contrast, high-dose (10-fold higher) ginseng extract decreased cell viability in both M5 and M30 cultures, even though total cell numbers and microglial counts remained unchanged. These results suggest that moderate concentrations of ginseng extract exert anti-inflammatory effects similar to isolated ginsenoside Rg1 by inhibiting pathological microglial proliferation, whereas high concentrations reduce cellular viability. This interpretation is supported by previous studies that reported enhanced astrocyte viability after treatment with higher concentrations of ginseng extract (1 mg/mL) for up to 48 h [41]. These different outcomes between our results and previous work may reflect variations in extract composition. The ginseng extract applied in our study may contain additional components that, at higher concentrations, could suppress cellular viability in astrocytes or microglia; however, the specific components responsible remain unclear.

In the untreated condition for both Rg1 and ginseng extract experiments, M30 cultures showed a higher proportion of RPT compared with M5 cultures. This suggests that the higher microglial density in M30 generated a more pathological environment and was associated with enhanced microglial activation. Consistent with this, untreated M30 cultures also exhibited increased cell viability compared with M5 cultures, reflecting increased microglial proliferation. This increase in cell viability in M30 cultures has also been reported in previous studies [15], though the underlying mechanism remains unclear. One explanation from other publications can be discussed as the involvement of the colony-stimulating factor 1 (CSF-1), a primary growth factor released by microglia, and its receptor CSF-1R, which are crucial for microglia survival and proliferation [42]. Consistent with this interpretation, CSF-1R inhibition has been shown to reduce microglial proliferation and activation during neuroinflammatory pathogenesis in a murine acute lipopolysaccharide model and experimental autoimmune encephalomyelitis model of multiple sclerosis [43]. These findings may explain why microglial counts and the proportion of RPT were higher in untreated M30 cultures compared with M5 cultures.

While treatment with Rg1 or ginseng extract did not result in significant changes in microglial phenotypes in physiological M5 cultures, in pathological M30 cultures, both Rg1 and moderate concentrations of ginseng extract significantly reduced the percentage of activated microglia (RPT) and increased the proportion of microglia exhibiting processes. Our results suggest that Rg1 and ginseng extract promote a shift in microglial phenotypes from activated to ramified/less activated or homeostatic (previously referred to as “resting”) states, thereby inhibiting activation of microglia. Previous studies described similar phenotypic transformations and showed that Rg1 ameliorated chronic mild stress (CMS) by transforming microglial phenotypes into a pro-neurogenic phenotype supporting hippocampal neurogenesis in male C57BL/6J mice [44]. The mechanism through which ginseng extract and Rg1 inhibit microglial activation and exert an anti-inflammatory effect could be multifactorial, according to previous research. Rg1 has also been reported to suppress hippocampal microglial activation in chronic social defeat stress mice and preventing depressive-like behavior via inhibition of NF-κB signaling [45]. In addition to shifting microglia from an activated to a homeostatic state, Rg1 has been shown to promote polarization from a pro-inflammatory to anti-inflammatory phenotype in a Parkinson’s disease mouse model [46]. In contrast to Rg1 and low-dose ginseng extract, high-dose ginseng extract did not induce significant phenotypical changes in microglia under either physiological or pathological conditions in our experiments. The lack of microglial response may reflect inhibition of microglial viability and a resulting devitalized cellular state, which could limit morphological responses to stimuli.

In previous studies, the proportion of activated microglia has been shown to be negatively correlated with astrocytic Cx43 expression, indicating that microglial activation may impact astrocytic intercellular communication in vitro [6]. Our study showed that Rg1 treatment under pathological conditions enhanced astrocytic gap-junctional intercellular communication and Cx43 expression, as assessed via immunocytochemistry, along with a reduction in the proportion of activated microglia. Interestingly, Cx43 expression showed a U-shaped pattern to Rg1 treatment, with a peak at 15 µM, a reduction at 30 µM and a subsequent increase at 45 µM. Although the mechanism underlying this none-linear dose–response pattern remains unclear, Cx43 expression remained higher at all Rg1 concentrations compared with the control group. Our findings are consistent with previous studies in primary cultured astrocytes and in rats showing that Rg1 ameliorates chronic unpredictable stress (CUS)-induced downregulation of Cx43 expression and fluorescence intensity, and the reduced Lucifer yellow diffusion distance caused by the gap-junction blocker carbenoxolone (CBX) [47]. Further, Rg1 has also been reported to ameliorate the impaired ultrastructural morphology of gap junctions, but not their function, under pathological conditions [47]. The inhibition of astrocyte Cx43 expression and intercellular communication by activated microglia could be attributed to their production of pro-inflammatory cytokines, which can influence Cx43. Microglial IL-1 beta and TNF- α were discussed to contribute to the inhibition of Cx43 expression [48]. The reversal of these effects by Rg1 may involve inhibition of microglial activation and pro-inflammatory cytokine production via multiple signaling pathways [45,49]. In addition, a previous study reported that Rg1 increases Cx43 protein levels via upregulation of Cx43 mRNA most probably by influencing ubiquitination and autophagy of Cx43 in rat primary astrocytes [50].

In contrast to Rg1, high-dose ginseng extract did not alter Cx43 expression, as measured by immunocytochemistry and immunoblotting, but an increased intercellular communication was observed using the scrape-loading–dye transfer method under pathological conditions. This discrepancy suggests that gap-junctional function does not directly correlate with total Cx43 expression. The enhanced intercellular communication may reflect a compensatory astrocytic response involving functional modulation of Cx43, such as changes in channel gating or membrane localization, rather than changes in protein expression levels [51,52]. Other studies have also demonstrated that the Cx43 channel blocker CBX can paradoxically increase Cx43 expression compared with Rg1-treated groups [47]. Further, cytokines released by microglia (IL-1 beta and TNF- α) oppositely regulate astrocytic Cx43 hemichannels and gap junction channels in vitro [53]. These observations emphasize the complexity of Cx43 regulation and suggest that future research should focus on investigating Cx43-related signaling mechanisms and their contributions to neuroinflammatory pathogenesis.

In comparison, isolated ginsenoside Rg1 exhibited more defined and consistent effects on microglial phenotypes and astrocytic coupling than ginseng extract. Both Rg1 and low-dose ginseng extract exerted anti-inflammatory effects under pathological conditions, including reduced microglial proliferation and activation, as well as in cases of Rg1 improved astrocytic gap-junctional intercellular communication and Cx43 expression. In contrast, the complex composition of ginseng extract resulted in more varied and dose-dependent effects, with high concentrations showing less specific modulation of microglial phenotypes and Cx43 expression.

## 5. Limitations

A limitation of our study is the lack of temporal controls for induction and neuroinflammation progression. In our study, microglial activation was triggered by increasing the microglial density in M30 co-cultures, which cannot mimic inflammatory progression from early to late phases. In other in vitro models, inflammatory stimulations such as LPS, corticosterone and interferon gamma have been employed to simulate acute and chronic inflammatory stages by adjusting the exposure duration [46,54,55,56,57,58]. Another limitation of this study is that the molecular mechanisms underlying the inflammatory processes, and their pharmacological modulation remain unclear. We discussed previously mentioned possible signaling pathways but cannot conclude these mechanisms from our experiments.

Previous studies have demonstrated that cytokine signaling regulates microglial phenotypes and astroglial coupling in co-culture models [7]. Future studies should examine the levels of pro- and anti-inflammatory cytokines following pharmacological treatment to better define the functional relationship between microglial activation and astrocytic coupling under inflammatory conditions. Further, we can only discuss viability related cellular effects without deeper differentiation like early cytotoxicity or apoptosis. Moreover, traditional visual analysis could be substituted with quantitative machine learning-based analysis in order to improve the accuracy of phenotypic assessments. Recent studies have demonstrated that machine learning-based morphometric analysis allows for the identification and analysis of microglial morphology based on morphometric parameters extracted through computational methods [59,60]. We must underline that due to different compositions a direct comparison between Rg1 and ginseng extract is only partially possible.

## 6. Conclusions

In conclusion, the present study demonstrated that ginseng and its bioactive component ginsenoside Rg1 exert anti-inflammatory effects in an astrocyte–microglia co-culture model of inflammation, suggesting their potential as complementary strategies for use alongside neuron-targeted CNS therapies. Specifically, Rg1 and low-dose ginseng extract reduced inflammation-induced microglial proliferation and activation, which were accompanied in cases of RG1 by enhanced astrocytic Cx43 expression and gap-junctional intercellular communication. In contrast, high-dose ginseng extract did not exert comparable effects on microglia or astrocytes and was associated with a higher reduction in glial cell viability compared to Rg1. These findings provide further insights into the anti-inflammatory properties of ginseng and highlight the importance of dose-dependent effects, particularly in defining the therapeutic window for targeting glial cell function to treat neuroinflammation.

## Data Availability

The raw data supporting the conclusions of this article are available on request from the corresponding author.

## Supplementary Materials

The following supporting information can be downloaded at: https://www.mdpi.com/article/10.3390/pharmaceutics18070806/s1. Supplementary S1: Chromatography of ginseng extract. Supplementary S2: Chromatography of the bulk of the ginseng extract. Supplementary S3: Certificate Graphical abstract created in BioRender.
