# Supplementary material for: Anti-Inflammatory Effects of Ginsenoside Rg1 and Low-Dose Ginseng Extract in an Astrocyte–Microglia Co-Culture Model of Inflammation
Source: Pharmaceutics. 2026 Jun 29;18(7):806. doi: 10.3390/pharmaceutics18070806 (PMC13415216; doi:10.3390/pharmaceutics18070806)
Supplement: Supplementary file 1 [file pharmaceutics-18-00806-s001.zip › Supplementary S2 Chromatography of the bulk of the ginseng extract.zip!/bulk.pdf]

Chromatograms

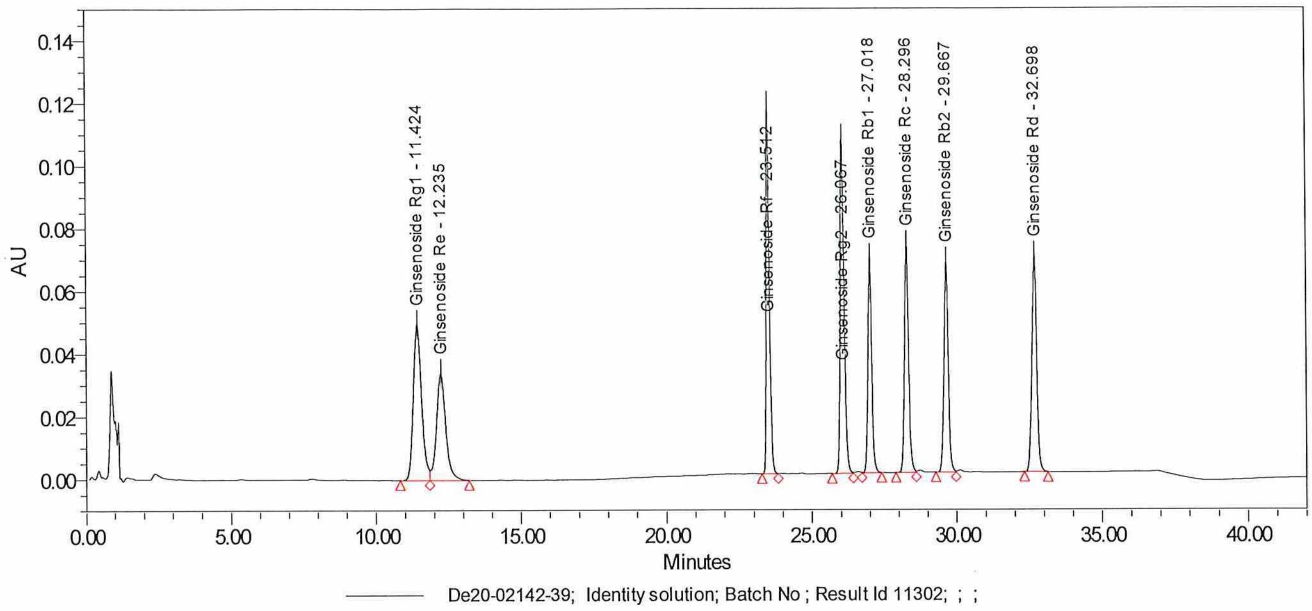

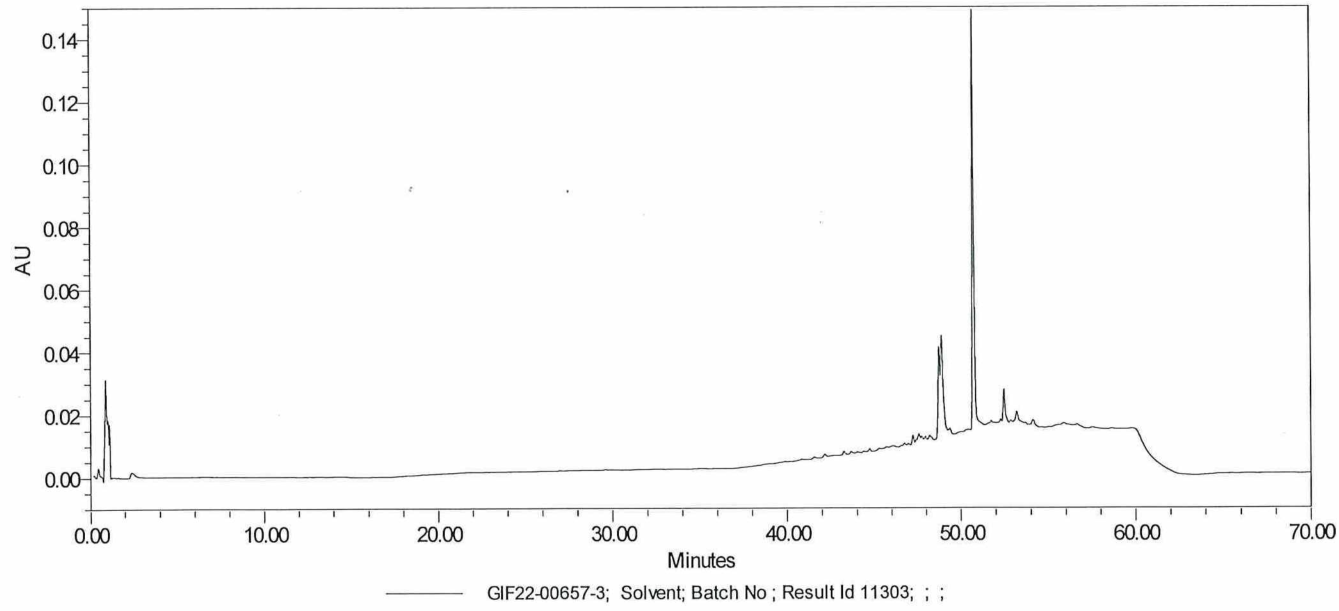

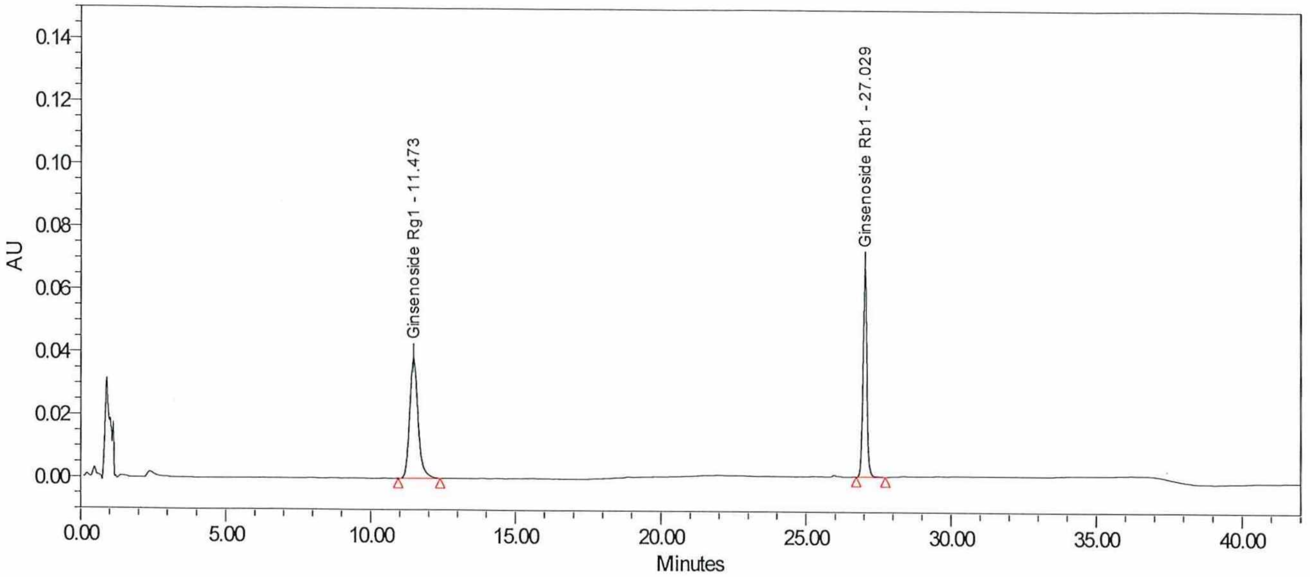

GIF22-00657-37; Reference solution 1; Batch No ; Result Id 11296; ; ;

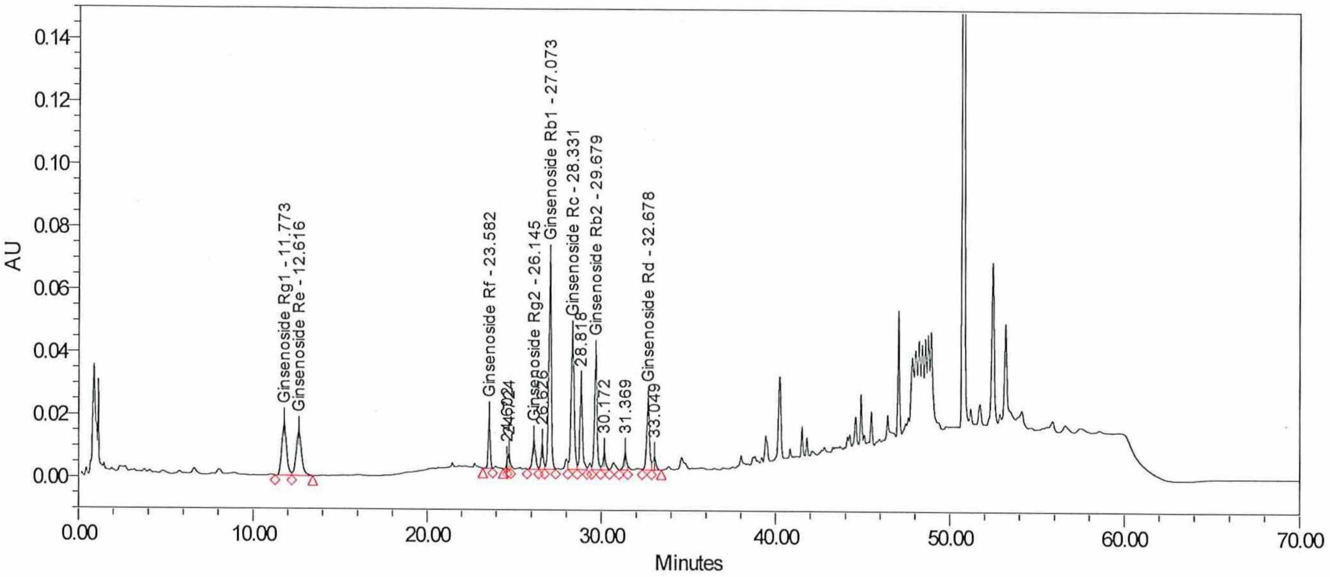

GIF22-00657-88; Roter Ginseng Pulverextrakt Kapseln; Batch No 0613782; Result Id 11304; ; ;

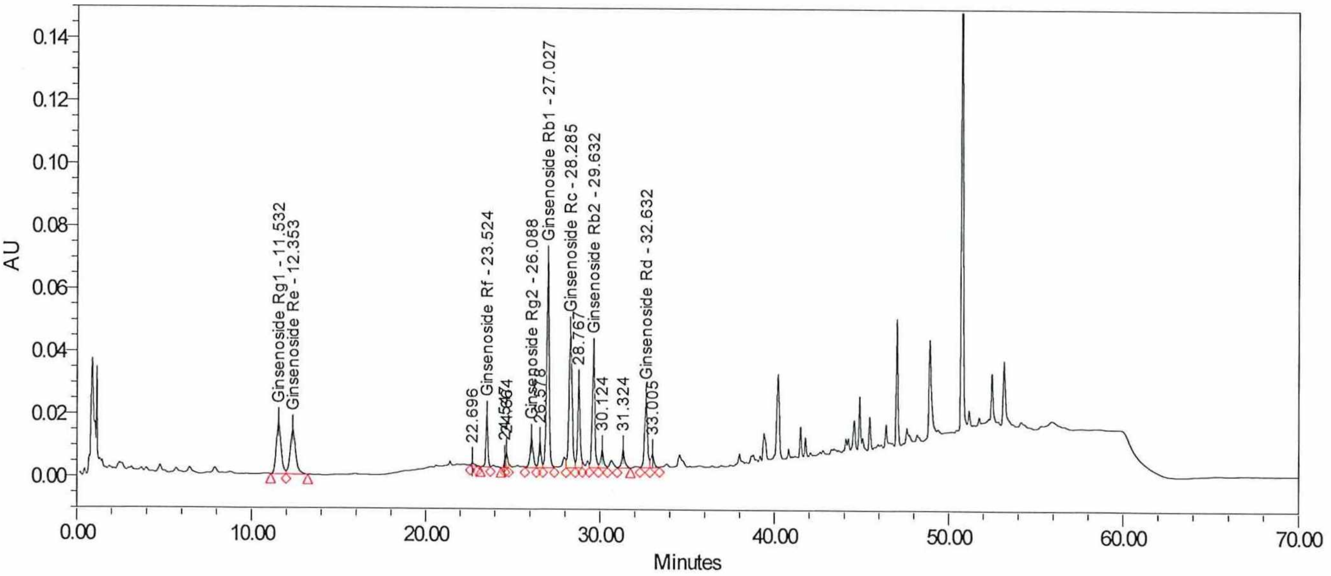

GIF22-00657-90; Rohstoff Ginseng; Batch No 0613782; Result Id 11308; ; ;

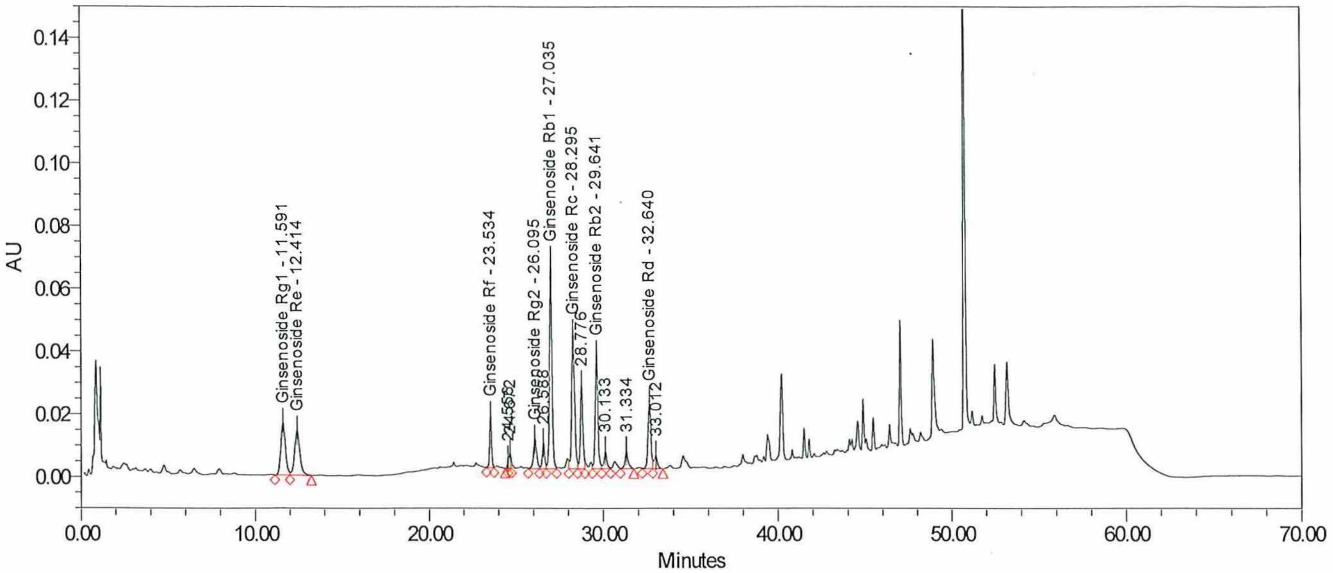

GIF22-00657-94; Roter Ginseng Extrakt Hartkapseln; Batch No 0613783; Result Id 11316; ; ;

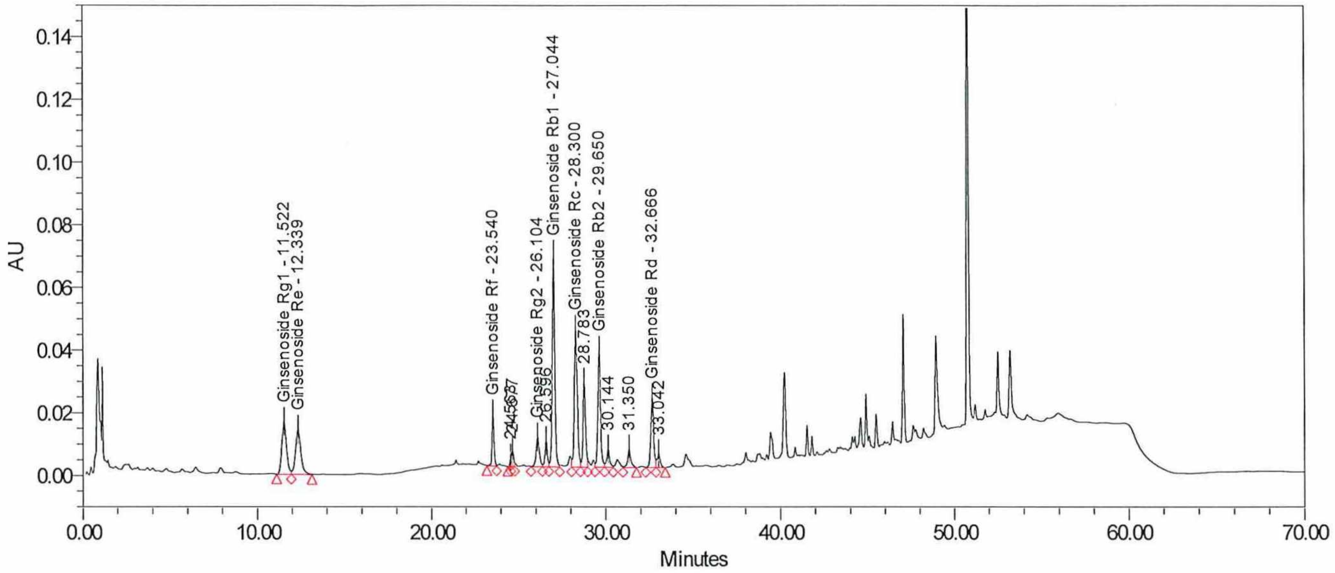

GIF22-00657-96; Rohstoff Ginseng; Batch No 0613783; Result Id 11320; ; ;

Fotodokumentation	HWI pharma services GmbH
Auftraggeber: 101257	Seite: 1 von 1
Auftrags-Nr.: AB-19-08223-1/18	

HWI group

Produkt: KRR Ginseng Pulverextrakt Kapseln und Roter Ginseng Extrakt Hartkapseln
Prüfdatum: 10.05.2022
Methode: AM 0533/2
Untersuchung: ☒ Freigabe ☐ Stabilität
Detektion bei: ☒ Tageslicht ☐ 254 nm ☐ 366 nm

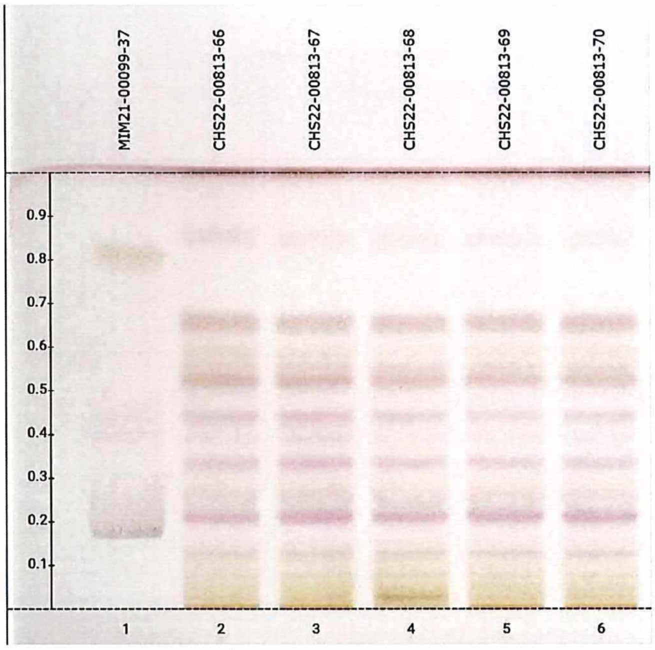

Nr.	Rohdaten-dokumentation	Charge	Laborcode	Bezeichnung / Lagerbedingungen
1	MIM21-00099-37	-	-	Identity solution
2	CHS22-00813-66	0613782	S220400070	KRR Ginseng Pulverextrakt Kapseln
3	CHS22-00813-67	0613782	S220400066	Trockenextrakt
4	CHS22-00813-68	0613782	S220400067	Trockenextrakt
5	CHS22-00813-69	0613783	S220400071	Roter Ginseng Extrakt Hartkapseln
6	CHS22-00813-70	0613783	S220400068	Trockenextrakt

Ergebnis: Das Chromatogramm entspricht hinsichtlich Lage, Intensität und Farbe dem Rohstoff.

12. MAI 2022 CHS

durchgeführt, Datum/Signum

18. MAI 2022 Wak

geprüft, Datum/Signum
