# Supplementary material for: Anti-Inflammatory Effects of Ginsenoside Rg1 and Low-Dose Ginseng Extract in an Astrocyte–Microglia Co-Culture Model of Inflammation
Source: Pharmaceutics. 2026 Jun 29;18(7):806. doi: 10.3390/pharmaceutics18070806 (PMC13415216; doi:10.3390/pharmaceutics18070806)
Supplement: Supplementary file 1 [file pharmaceutics-18-00806-s001.zip › Suppllementary S1 Chromatography of ginseng extract.zip!/extract chromatography.pdf]

Fotodokumentation | **HWI pharma services GmbH**

**Auftraggeber:**	**10002**	**Seite:**	1 von 1
**Auftrags-Nr.:**	**AB-19-08223-1/16**		

HWI group

Produkt: Trockenextrakt aus Roter Ginsengwurzel

Prüfdatum: 08.03.2022

Methode: EAM 0914_1-0

Untersuchung: ☒ Freigabe ☐ Stabilität

Detektion bei: ☒ Tageslicht ☐ 254 nm ☐ 366 nm

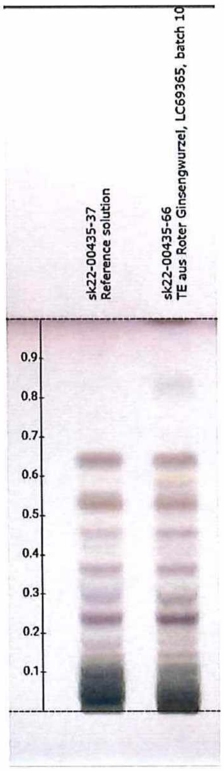

Nr.	Rohdaten-dokumentation	Charge	Laborcode	Bezeichnung / Lagerbedingungen
1	sk22-00435-37	-	-	Referenzlösung Ginseng TE HRS
2	sk22-00435-66	10000287/01	69365	Trockenextrakt aus Roter Ginsengwurzel

Ergebnis: Das Chromatogramm entspricht hinsichtlich Lage, Intensität und Farbe den Vorgaben der Ph. Eur. Monografie.

08. MRZ. 2022 [signature]
durchgeführt, Datum/Signum

24. MRZ. 2022 [signature]
geprüft, Datum/Signum

Chromatograms

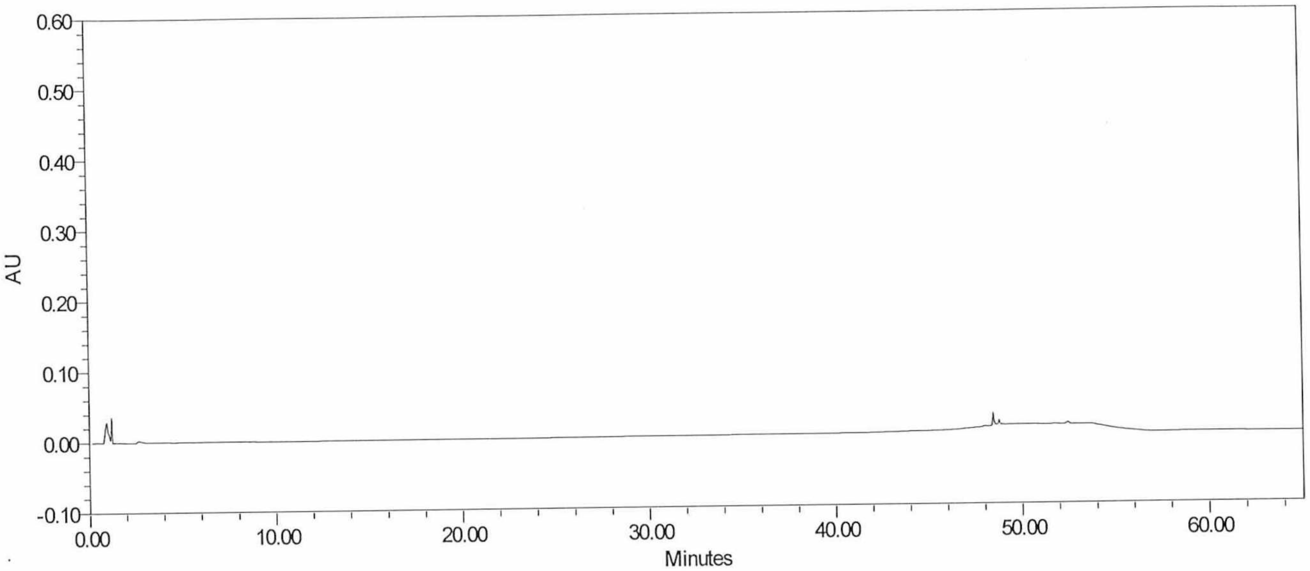

sv22-00327-3; Solvent; LabCode ; Batch No ; Result Id 2695; ; ;

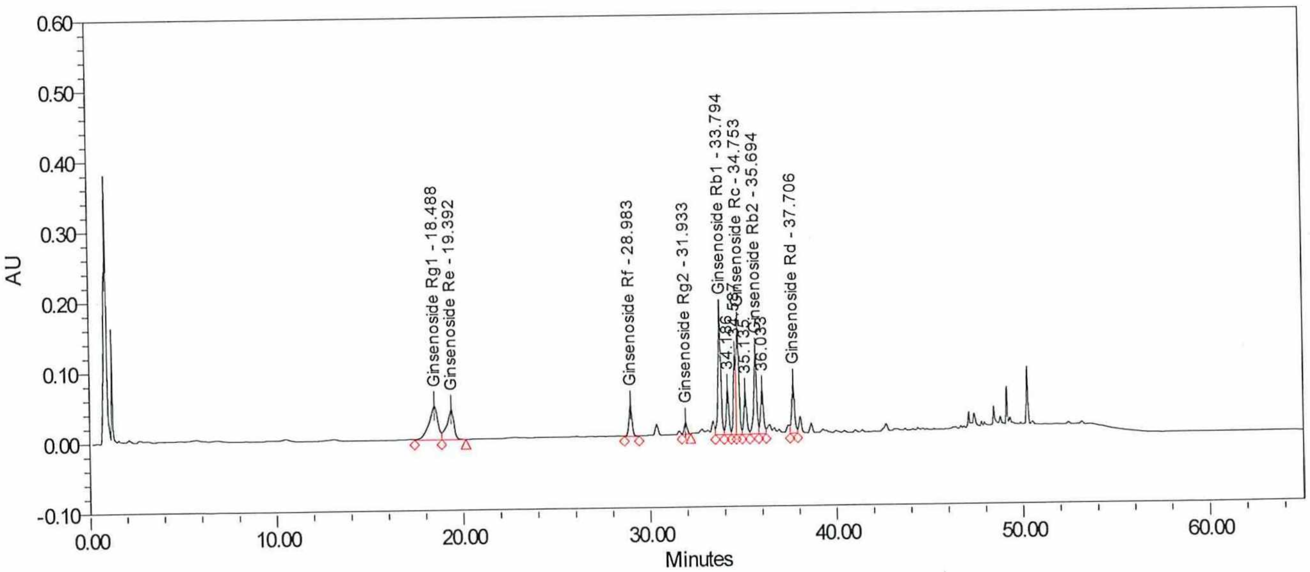

sv22-00327-90; Reference solution a; LabCode ; Batch No ; Result Id 2740; ; ;

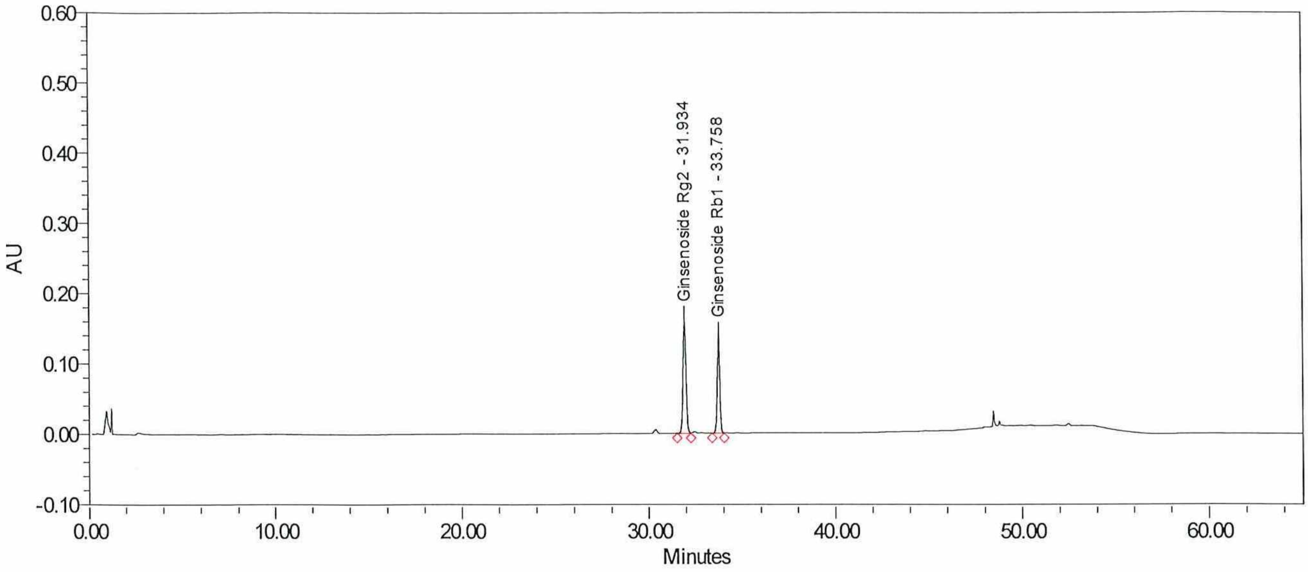

sv22-00327-41; Reference solution d; LabCode ; Batch No ; Result Id 2699; ; ;

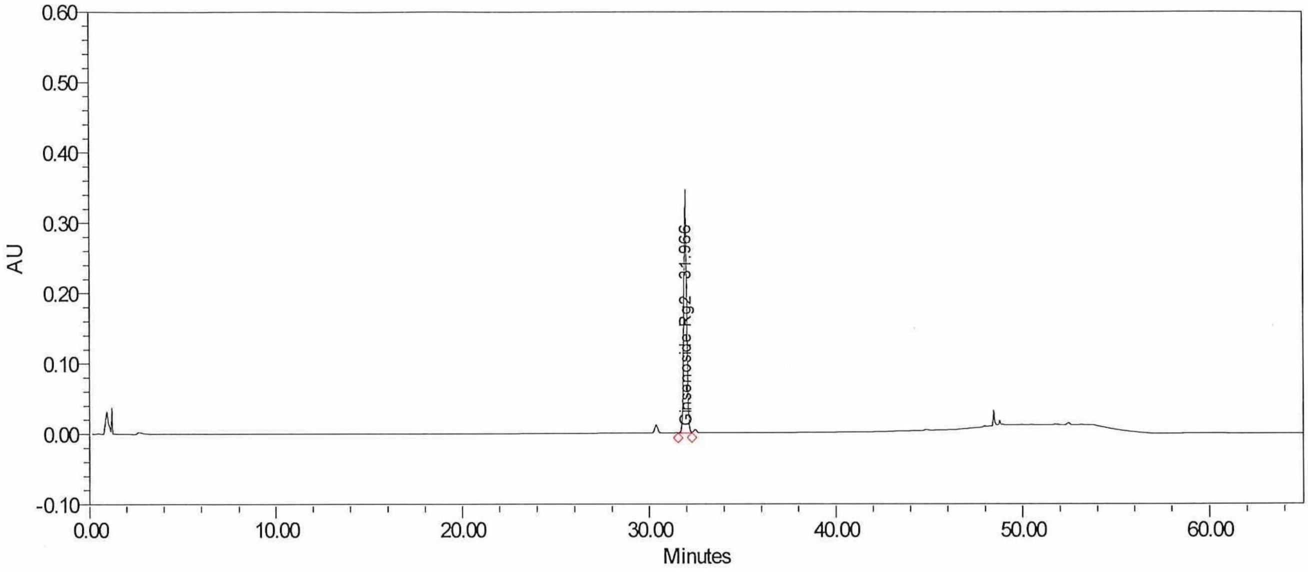

sv22-00327-40; Reference solution c; LabCode ; Batch No ; Result Id 2700; ; ;

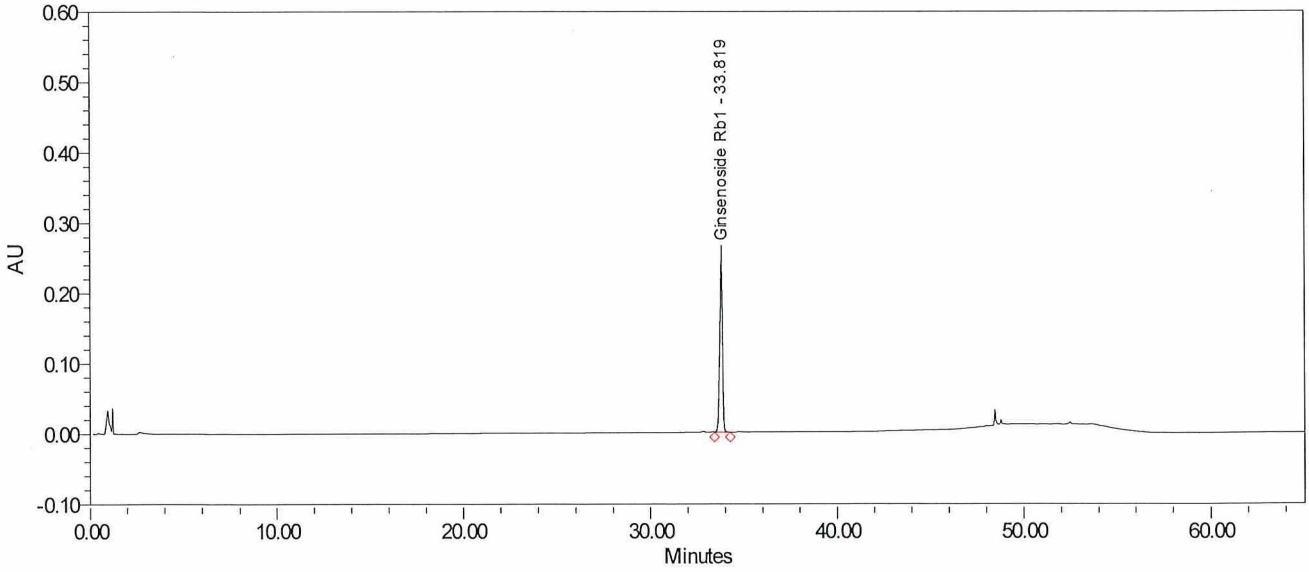

sv22-00327-38; Reference solution b - 1; LabCode ; Batch No ; Result Id 2688; ; ;

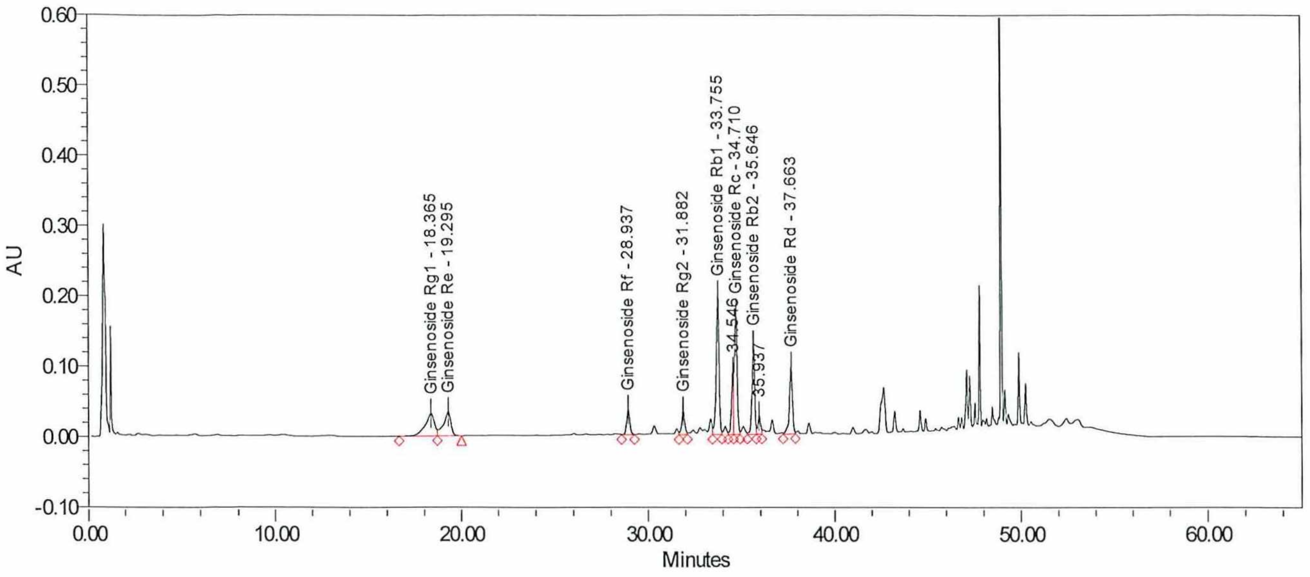

sv22-00327-80; Ginseng dry extract; LabCode 69365; Batch No 10000287/01; Result Id 2703; ; ;
